# Dermoscopic Features of Psoriatic Nails and Their Correlation to Disease Severity

**DOI:** 10.1155/2023/4653177

**Published:** 2023-05-15

**Authors:** Zeinab R. Mashal, Emad Eldin A. Elgamal, Mohamed S. Zaky, Mohamed L. Elsaie

**Affiliations:** ^1^Damietta Dermatology and Leprosy Hospital, Ministry of Health and Population, Damietta, Egypt; ^2^Department of Dermatology, Venereology and Andrology, Damietta Faculty of Medicine, Al-Azhar University, Cairo, Egypt; ^3^Department of Dermatology, Medical and Clinical Research Institute, National Research Centre, Cairo, Egypt

## Abstract

**Background:**

Nail psoriasis is a challenging chronic condition affecting patients functionally and psychologically. Nail involvement is observed in 15–80% of psoriatic patients with occasional presence of isolated nail psoriasis.

**Objectives:**

To evaluate dermoscopic features of nail psoriasis and correlate them clinically.

**Methods:**

The study included fifty subjects with nail psoriasis. Psoriasis skin and nail severity was evaluated using psoriasis area severity index (PASI) and nail psoriasis severity index (NAPSI). Dermoscopy of the nails (onychoscopy) was performed, and features were recorded and analyzed.

**Results:**

The most common clinical and dermoscopic findings were pitting (86%) and onycholysis (82%). Among all dermoscopic features of nail psoriasis, only longitudinal striations and subungual hyperkeratosis were significantly higher in patients with moderate to severe psoriasis than in patients with mild psoriasis (*P*=0.028; *P*=0.042, respectively). PASI scores correlated positively but none significantly with NAPSI scores (*r* = 0.132, *P*=0.360), and similarly, no significant correlation was observed between the duration of psoriasis and the dermoscopic NAPSI (*r* = 0.022, *P*=0.879).

**Conclusion:**

Dermoscopy can serve as a useful tool for early diagnosis of psoriatic nail changes not always visible to the naked eye and is a non-invasive easy-to-use confirmatory tool for nail changes in psoriatic disease or in isolated nail involvement.

## 1. Introduction

Psoriasis represents a chronic skin condition affecting 1-2% of world population [1]. Furthermore, nail involvement in psoriasis affects approximately 15–80% of cases. Only 5% of psoriatic patients present with isolated nail changes with no skin involvement [2].

A wide range of psoriatic nail clinical presentations can be identified based on the site of anatomical affection (nail matrix/nail bed) [3]. Nail matrix involvement presents as pitting, leukonychia, and crumbling as well as predominant red spots that can be seen in the lunula. On the other hand, nail bed affection can manifest with an array of presentations as subungual hyperkeratosis, splinter hemorrhages, and onycholysis [4].

Far beyond a cosmetic disfigurement, nail involvement also leads to substantial disease burden as well as impaired daily activity and stigmatization. Moreover, nail affection can be a marker of disease severity and a prognostic factor for disease progression into psoriatic arthritis [5].

Diagnostic biopsy remains to be the gold standard of diagnosing nail psoriasis, yet it remains to be painful. Dermoscopy is a non-invasive tool whose scope has extended beyond its initial use in cutaneous melanoma to become an adjunctive tool in diagnosing many pigmented and non-pigmented skin diseases including nail disorders which is termed as onychoscopy [4, 5].

The aim of this study was to evaluate the dermoscopic nail features in psoriatic nails, comparing the dermoscopic examination with clinical examination to determine the correlation between the dermoscopic nail features and the disease severity using psoriasis area severity index (PASI) score and nail psoriasis severity index (NAPSI) score.

## 2. Patients and Methods

This cross-sectional study was performed during the period between April 2021 and December 2021 on fifty (50) patients diagnosed clinically with psoriasis with nail involvement to detect the dermoscopic nail features among them. Ethical committee approvals were obtained in advance, and included patients were instructed on the study procedures and consented if willing to participate. The study was approved by the Ethics Committee of Damietta Faculty of Medicine IRB (00012367-22-34-110), Al-Azhar University, Egypt.

Patients who fulfilled inclusion crieteria of the study had a full personal and clinical history examination. Demographic data, disease onset and duration, and any associated comorbidities were all recorded. KOH mounts obtained from nail clippings were performed to diagnose and exclude any subjects with onychomycosis. Patients complaining of erythrodermic form of psoriasis or suffering from systemic diseases affecting nails were excluded. Similarly, pregnant or lactating females were excluded from the study.

To determine psoriasis disease severity, the whole body was divided into four sections (head (H) (10% of a person's skin); arms (A) (20%); trunk (T) (30%); and legs (L) (40%)) that when added up provided a final PASI score. A score of 72 is the highest that could be achieved and demonstrates a 100% body affection with psoriasis while zero (0) score is the lowest that can be achieved and demonstrates no skin involvement. PASI scores can be divided into mild (below 10); moderate (10–20); and severe if above 20 [6].

The NAPSI scoring system was used to evaluate psoriatic nail affection after thorough clinical and dermoscopic evaluation of nails. To calculate NAPSI, each nail was divided into 4 equal and symmetrical quadrants to properly assess nail matrix and nail bed changes. Each nail matrix and nail bed change was given a score of 1 if present and zero (0) if absent. A score of 1 is given for the presence of such signs in every quadrant, so that there is a nail matrix score of 0–4 and nail bed score of 0–4 per nail with a minimum score of 0 and a maximum score of 8 per nail. Any additional nail changes were also recorded [5].

Dermoscopic images were captured with a DermLite DL4, ×10 dermoscope using polarized and non-polarized modes. Digital photography using the mobile camera (48MP) was used for recording micro- and macroimages.

### 2.1. Statistical Analysis

Following the conduction of the study and provision of results, analysis was reproduced using SPSS 27 package. Standard deviation, median data, and Pearson chi-square descriptive statistics were calculated and analyzed and summarized to compare between the different groups on basis of qualitative variables provided and reproduce any statistical significance. Fisher's test was used instead of chi-squared (*χ*^2^) test when the assumption that at least 80% of the expected frequencies are greater than five was violated. On the other hand, correlation analysis was reproduced using the Spearman coefficient (rs) to indicate and highlight any significant association of variables used.

## 3. Results

This study included 50 patients with psoriasis. Their mean age was 44 ± 18.62 years. Among the cases, 41 were males (82%) and 9 were females (18%). The mean duration of the disease was 11.78 ± 11.14 years, and the median of cutaneous psoriasis duration was 9 years.

The mean PASI score in the included cases was 11.57 ± 7.78 while the mean NAPSI score in the included cases was 30.66 ± 13.67. Twenty-three cases (46%) suffered from mild psoriasis while 27 cases (54%) complained of moderate to severe activity (Table 1).

**Figure 1:**
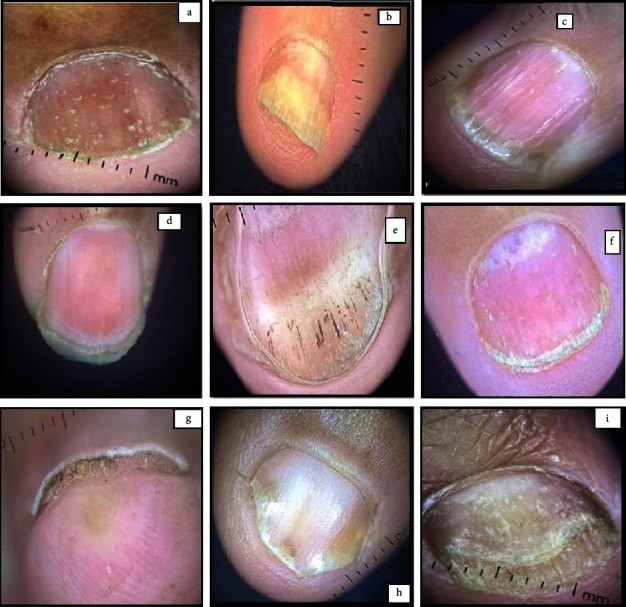
Various manifestations of nail psoriasis (NP). (a) Pitting; (b) onycholysis; (c) longitudinal striations; (d) pitting along with striations and subungual keratosis (e) splinter hemorrhages; (f) red lunula spotting; (g) subungual hyperkeratosis; (h) salmon patches; (i) onycholysis with distal plate crumbling.

The dermoscopic features in the study cases included pitting in 43 cases (86%), onycholysis in 41 cases (82%), longitudinal striations in 32 cases (64%), prominent capillaries at the onychodermal band in 28 cases (56%), splinter hemorrhages in 26 cases (52%), salmon patch in 23 cases (46%), subungual hyperkeratosis in 23 cases (46%), leukonychia in 13 cases (26%), Beau's lines in 12 cases (24%), spotted lunula in 5 cases (10%), crumbling in 5 cases (10%), onychomadesis in 3 cases (6%), melanonychia in 2 cases (4%), and transverse striations in 1 case only (2%) (Table 2; Figure 1).

When checking all dermoscopic features of nail psoriasis recorded, only the longitudinal striations and subungual hyperkeratosis were significantly higher in subjects with moderate to severe psoriasis when compared to those with mild psoriasis (*P*=0.028; *P*=0.042, respectively) (Table 3).

**Figure 2:**
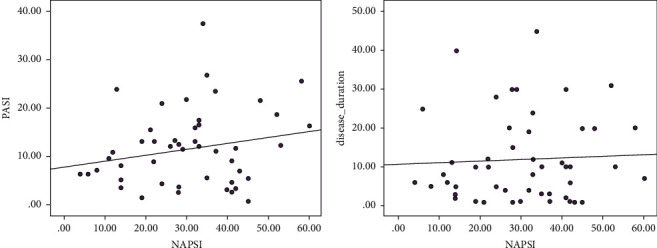
(a) Correlation between NAPSI and PASI scores. (b) Correlation between NAPSI and disease duration.

PASI scores correlated positively but none significantly with NAPSI scores (*r* = 0.132, *P*=0.360), and similarly, no significant correlation was observed between the duration of psoriasis and the dermoscopic NAPSI (*r* = 0.022, *P*=0.879) (Figure 2).

## 4. Discussion

Dermoscopy is a non-invasive easy-to-use tool used for diagnosis and follow-up of many skin diseases [7]. Despite histopathology being the golden diagnostic tool in psoriasis, dermoscopy of nails (onychoscopy) can serve as an efficient tool for visualisation, confirmation, and follow-up of patients with nail psoriasis [8].

So far, there is a paucity of studies observing the features of nail psoriasis and correlating them to disease severity and there is no consensus for using this efficient tool in diagnosing and follow-up of nail psoriasis lesions [9].

Pitting (*n* = 43; 86%) was the most common dermoscopic finding observed in the current study. Pitting can be appreciated and seen clinically as shallow dents on the nail plate which form due to parakeratosis of the matrix while dermoscopically, it can be seen as punctuate dents surrounded by a white hue [2]. Within the same context, Khopkar and Yadav [10] and Chauhan et al. [11] disclosed that pitting was the commonest of all dermoscopic features seen in their relevant studies. Moreover, another study determined pitting to be the most reliable and third most common clinical finding among psoriatic patients [2] (Table 4).

Separation of the nail plate and nail bed is often referred to as onycholysis [12]. A linear erythematous border around the onycholytic areas can be specifically seen by dermoscope which is rarely observed by the naked eye and can be considered a specific onycholysis finding in psoriatic nails [13].

In the current study, onycholysis was the second most common finding and detected in 41 cases (82%). In agreement with our findings, Khopkar and Yadav [10] showed that onycholysis was the second most dermoscopic finding in patients suffering from nail psoriasis and recorded the observation in 10 out of 46 patients. Also, Polat and Kapıcıoğlu [2] determined the incidence of onycholysis by dermoscopy to be 77.5%, ranking the third most common finding among their study participants. Long et al. [14] reported onycholysis to be the commonest finding among their study participants while Wanniang et al. [4] discovered onycholysis in 54% of cases suffering from nail psoriasis in their study.

Salmon patches, also known as the oil drop sign, refer to the yellowish-red discoloration that appear as irregular translucent areas visible through the nail plate. In the current study, salmon patches were a common finding and seen in 23 cases (46%). This was in accordance with other studies that dermoscopically observed salmon patches in 47.5% and 44% of patients, respectively [2, 4].

In the current study, longitudinal striations were reported in 32 cases (64%). This was in agreement with Chauhan et al. who showed that longitudinal ridging could be appreciated in 57.3% of fingernails and 22.7% toenails [11].

Splinter hemorrhages were dermoscopically detected in 52% of cases in this study while a higher incidence of 62%, 73.1%, and 80% was reported by other authors [2, 4, 5].

Leukonychia is due to the inclusion of parakeratotic cells in the nail from lesions in the matrix [15]. In the current study, leukonychia was reported in 13 cases (26%) similar to the finding of Waaniang who detected leukonychia among 22% of their studied cases (*n* = 50) [4].

Subungual hyperkeratosis was the second most common nail bed dermoscopic finding in our study and significantly correlated with disease severity (*P*=0.042) while in the study conducted by Chauhan et al., it was the most common nail bed dermoscopic finding in both fingernails and toenails [11].

In our study, a red lunula was detected in only 5 cases (10%). Long et al. [14] significantly related red lunula and the presence of agminated capillary dots to dilated streaky capillaries (*χ*^2^ = 6.51, *P* < 0.05; *χ*^2^ = 9.83, *P* < 0.01, respectively) [14].

In the current study, crumbling was noted in 5 cases (10%) while in the study conducted by Chauhan et al. [11], crumbling (22.79%) was the fifth nail matrix dermoscopic finding after pitting, longitudinal ridging, fuzzy lunula, and leukonychia, respectively. Other studies reported crumbling dermoscopically in 10%, 16%, and 22.2%, respectively [2, 4, 14].

In the current study, there was a weak non-significant correlation between NAPSI and PASI (*r* = 0.132, *P*=0.360). Our findings matched findings by Wanniang, Long, and Arora who reported a positive correlation between the total NAPSI score and PASI score (*r* = 0.535, *P* < 0.001; *r* = 0.9013, *P* < 0.05; *r* = 0.56, *P* < 0.001, respectively) [4, 15, 16]. Such finding anticipates that nail affection is probably higher with severe forms of systemic inflammation; however, this could be biased by the fact that some dermoscopic features of nail affection still persist after remission of activity in some patients [4, 15, 16].

The current study demonstrated a weak non-significant correlation between NAPSI and psoriasis duration (*r* = 0.022, *P*=0.879). In the study conducted by Wanniang et al., a weak correlation was observed between the duration of psoriasis and the dermoscopic NAPSI score (*r* = 0.2835; *P*=0.046). They speculated that duration of cutaneous psoriasis is associated with longer and more severe affection of nail psoriasis [4]. No statistically significant difference was noted in the distribution of nail psoriasis between the cases with mild psoriasis and cases with moderate to severe psoriasis except for longitudinal striations and subungual hyperkeratosis which were significantly higher in the moderate to severe psoriasis group (*P*=0.028; *P*=0.042, respectively).

To date, reports on dermoscopic features of nail psoriasis in relation to disease severity were few and the existing data were not conclusive. In one study, dilated capillaries, thickening of the nail plate, subungual hyperkeratosis, and pitting were found to be associated with higher disease severity [17] while in another study, red lunula and longitudinal fissures were more relevant to the severity of psoriasis [15].

The small study sample represents a limitation besides the inability to investigate the dermoscopic findings of nail psoriasis in other subtypes of psoriasis not included in this study. Lack of toe nail assessment represents another limitation of the study as well as the male to female pattern of inclusion. Furthermore, there was no control and no assessment of dermoscopic changes after treatment.

In conclusion, dermoscopy can serve as a useful tool for early diagnosis of psoriatic nail changes not always visible to the naked eye and is a non-invasive easy-to-use confirmatory tool for nail changes in psoriatic disease or in isolated nail involvement.

A hallmark advantage of dermoscopy is its ability to interface between histopathologic and clinical examinations as well as its ability to aid in early diagnosis of nail affection even before clinical signs are evident. Taking into consideration this advantage of early dermoscopic features in clinically uninvolved nails, dermoscopy can be used as a marker of disease activity and progression. This study comprehensively describes the dermoscopic features of different parts of nail unit in patients of nail psoriasis. Larger sample size and evaluation of different types of psoriatic nail changes before and after treatment are required to elucidate distinct relationships between dermoscopic changes and disease severity.

## Figures and Tables

**Table 1:** Analysis of the disease severity in the psoriasis group.

Severity index		Study cases (*n* = 50)
PASI	Mean ± SD	11.57 ± 7.78
	Median (min-max)	11.35 (0.9–37.5)

NAPSI	Mean ± SD	30.66 ± 13.67
	Median (min-max)	32 (4–60)

*Psoriasis severity*		
Mild		23 (46%)
Moderate to severe		27 (54%)

Continuous data are expressed as mean ± SD and median (range). Categorical data are expressed as number (%).

**Table 2:** Analysis of the dermoscopic features in the psoriasis cases.

Dermoscopic feature	Study cases (*n* = 50)
Pitting	43 (86%)
Onycholysis	41 (82%)
Longitudinal striations	32 (64%)
Prominent capillaries at onychodermal band	28 (56%)
Splinter hemorrhage	26 (52%)
Salmon patch	23 (46%)
Subungual hyperkeratosis	23 (46%)
Leukonychia	13 (26%)
Beaus lines	12 (24%)
Spotted lunula	5 (10%)
Crumbling	5 (10%)
Onychomadesis	3 (6%)
Melanonychia	2 (4%)
Transverse striations	1 (2%)

Categorical data are expressed as number (%).

**Table 3:** Analysis of dermoscopic features in cases according to severity.

Dermoscopic feature	Group 1 (mild psoriasis) (*n* = 23)	Group 2 (moderate to severe psoriasis) (*n* = 27)	Test of significance
Pitting	19 (82.6%)	24 (88.9%)	*χ* ^2^ = 0.407
			*P*=0.527

Onycholysis	17 (73.9%)	24 (88.9%)	*χ* ^2^ = 1.887
			*P*=0.170

Longitudinal striations	11 (47.8%)	21 (77.8%)	*χ* ^2^ = 4.836
			*P*=0.028^*∗*^

Prominent capillaries	11 (47.8%)	17 (63%)	*χ* ^2^ = 1.155
			*P*=0.283

Splinter hemorrhage	14 (60.9%)	12 (44.4%)	*χ* ^2^ = 1.342
			*P*=0.247

Salmon patch	10 (43.5%)	13 (48.1%)	*χ* ^2^ = 0.109
			*P*=0.741

Subungual hyperkeratosis	7 (30.4%)	16 (59.3%)	*χ* ^2^ = 4.154
			*P*=0.042^*∗*^

Leukonychia	4 (17.4%)	9 (33.3%)	FET = 1.641
			*P*=0.200

Beaus lines	5 (21.7%)	7 (25.9%)	*χ* ^2^ = 0.119
			*P*=0.730

Spotted lunula	4 (17.4%)	1 (3.7%)	FET = 2.585
			*P*=0.108

Crumbling	3 (13%)	2 (7.4%)	FET = 0.438
			*P*=0.508

Onychomadesis	1 (4.3%)	2 (7.4%)	FET = 0.206
			*P*=0.650

Melanonychia	1 (4.3%)	1 (3.7%)	FET = 0.013
			*P*=0.908

Transverse striations	1 (4.3%)	0 (0%)	FET = 1.198
			*P*=0.274

*P*: probability. Categorical data are expressed as number (%). *χ*^2^: chi-square test. FET: Fisher's exact test. ^*∗*^Statistically significant (*P* < 0.05).

**Table 4:** Comparison of nail dermoscopic features with similar studies on nail changes in psoriasis.

Nail features	Bindagi et al. [8]		Wanniang et al. [4]		Polat et al. [2]		Yadav and Khopkar [9]	Our study
	Clinical	Onychoscopy	Clinical	Onychoscopy	Clinical	Onychoscopy	Onychoscopy	Onychoscopy
	(%)	(%)	(%)	(%)	(%)	(%)		
	Sample size: 60		Sample size: 50		Sample size: 40		Sample size: 68	Sample size: 50
*Nail matrix changes*								
Pitting	90	95	84	84	92.5	77.5	18	86
Leukonychia	48.33	78.33	20	22	82.5	92.5	—	26
Crumbling of nails	55	53.33	14	16	17.5	20	—	10
Red spots in lunula	20	30	0	8	5	5	—	10

*Nail bed changes*								
Subungual HK^*∗*^	81	76.67	40	46	35	32.5	—	46
Onycholysis	55	80	54	54	67.5	77.5	10	82
Splinter Hgs^*∗*^	30	61.67	8	62	75	80	5	52
Salmon patch	18.33	23.33	32	44	42.5	47.5	2	46

^*∗*^Subungual HK: subungual hyperkeratosis; splinter Hgs: splinter hemorrhages.

## Data Availability

The data used to support the findings of this study are available from the corresponding author upon request.
